# Pediatric Dental Appointments No-show: Rates and Reasons

**DOI:** 10.5005/jp-journals-10005-1506

**Published:** 2018-06-01

**Authors:** Rupinder Bhatia, Esha C Vora, Anup Panda

**Affiliations:** 1 Professor and Head, Department of Pediatric and Preventive Dentistry, D. Y. Patil School of Dentistry, Navi Mumbai, Maharashtra, India; 2 Postgraduate Student, Department of Pediatric and Preventive Dentistry, D. Y. Patil School of Dentistry, Navi Mumbai, Maharashtra, India; 3 Professor, Department of Pediatric and Preventive Dentistry, D. Y. Patil School of Dentistry, Navi Mumbai, Maharashtra, India

**Keywords:** Broken appointments, Missed appointments, No-shows.

## Abstract

**Aim:**

To determine the frequency and reasons of missed and canceled pediatric dental appointments and identifying the factors associated with it among the patients visiting the pediatric dental clinic of the dental college in Navi Mumbai, Maharashtra, India. To assess the parents’ perception regarding the treatment of their children and to explore the merit of different management strategies regarding the missed appointments.

**Materials and methods:**

Self-administered questionnaires were handed over to the parents in the waiting area, whose children were undergoing treatment in the pediatric dental clinic. Questionnaire included questions pertaining to the sociodemo-graphic characteristics, methods of remembering appointments, and satisfaction with the overall past clinic experience.

**Results:**

Of the 294 surveyed sample, 52.0% stated that they have missed an appointment. Highest percentage of the patients had missed due to parents’ forgetfulness and patient’s exams; 52.0% relied on their memory to remember their appointments, and 44.5% used their diaries and mobiles as a means of reminders. For canceling an appointment, most of them stated that patient was unwell and parents’ commitments toward other work led to an increase in rates of no-shows. Socioeconomic status and the methods of remembering the appointment date were found to be significantly correlated with missing an appointment among the surveyed sample (p < 0.05); 48.3% said that they would want a call from the dentist a day prior to their appointment as a reminder.

**Conclusion:**

Around one half of the patients attending the pediatric dental clinic missed their appointments. Patients most likely to fail to keep an appointment was the one who was young, remembered appointment via memory, came from a high socioeconomic class, most of them were males, and had previously broken appointments, while patients preferred receiving a call from the dentist as a reminder aide.

**Clinical significance:**

Missing appointment creates a series of problems. Firstly, it deprives another patient the opportunity of getting treated. Secondly, it contributes to the development of long waiting list for dental services. Thirdly, it affects the patient’s treatment time and may cause increase in the dental emergencies leading to a disruption in the doctor-patient relationship and behavioral management problems.

**How to cite this article:** Bhatia R, Vora EC, Panda A. Pediatric Dental Appointments No-show: Rates and Reasons. Int J Clin Pediatr Dent 2018;11(3):171-176.

## INTRODUCTION

“An appointment missed by you is an appointment missed by two” was rightly said by Capt Kim Decker, chief of MACH’s Health care Management Division. It means that if you miss the first appointment, you are likely going to need another appointment, and then whoever did not get an appointment initially is also still needing one.^1^

A missed appointment is defined as an appointment for which the patient did not show up, did not call in to cancel or reschedule. Improving treatment proficiency and office output, through well-structured practices, should be the goal of every clinician. This, however, is affected by patient’s nonattendance which is a significant problem and area of concern for health care providers.^2^

Access to oral health care is jeopardized when patients miss scheduled appointments.^3^ Missed appointments affect patients, as it leads to loss of continuity of care; this in turn could cause a health risk that might eventually contribute to an increase in the emergency room visits or chronic conditions.^4^ No-shows affect the productivity and efficiency of the dental care facility. It also causes disruption of the health care delivery system for other patients. A study conducted in USA (2005) revealed that missed appointment was the most significant factor related to treatment failure.^5^

Missing and canceling the appointment preclude other eligible patients from receiving care they deserve, exert a huge strain on the system and prolong booking and waiting time in the clinic for other patients of earlier appointments which affect their quality of care.^5^ When a patient misses his/her dental appointment, that patient is denying himself/herself dental care which is beneficial to them if not urgent.^6^ Economically, missed appointment has significant impact on the ability to provide efficient and effective outpatient services. It results in wasting financial and social costs. The social cost is the costs of unused or misused resources, such as clinic capacity and possible misuse of patients’ time.^5^ This puts an extra load on the health care system and costs the clinics a large amount of money and contributes to depletion of limited resources.

According to Detman and Gorzka,^3^ a missed appointment is likely to be affected by three kinds of barriers: Personal, structural, and financial. Personal barriers consist of factors like attitude toward oral health care, education level, and various demographic characteristics. Structural barriers, such as transportation, clinic hours, and the way providers organize their services can also impede access to appointments. Various financial barriers can also affect a patient’s ability to keep appointments.

Till date, no work has been undertaken to investigate the attendance pattern of patients in pediatric dental clinic in Navi Mumbai, Maharashtra, India. So the aim of the study was to determine the frequency and reasons of missed and canceled pediatric dental appointments and any association between their nonattendance and some demographic variables among patients undergoing active treatment in the pediatric dental clinic in the dental college in Navi Mumbai, Maharashtra, India. In addition, the aim was to assess the parents’ perception regarding the treatment of their children, to find out the methods by which patients remembered their appointments, and to explore the merit of different management strategies and create awareness regarding the same.

## MATERIALS AND METHODS

Self-administered questionnaires were designed in English which was then translated into the regional language. These questionnaires were handed over to the parents in the waiting area whose children were undergoing treatment during the period of 6 months from January 2015 to July 2015. The questions were filled by the parents and required several category choices or a range from very good to very bad. These questionnaires were then tested on subjects and improvements were made according to the feedback. A total of 294 samples were collected. All questionnaires were checked for completeness before they were collected.

The questionnaire consisted of the following questions pertaining to the:

- Sociodemographic characteristics,
- Methods of remembering appointments,
- Reasons for canceling and missing appointments and
- Satisfaction with the overall past clinic experience.

Data were analyzed using six variables in relation to the missed appointments:

1. Age of the child
2. Gender of the child
3. Parent’s education status
4. Socioeconomic status of the family
5. The reason for canceling the appointment
6. The methods to remember the appointment date

Kendell’s Tau-B test was performed to test for the correlation. All tests were conducted at 0.05 significance level.

## RESULTS

A total of 294 completely filled questionnaires were collected and analyzed.

Of the surveyed sample, nearly half the participants stated that they had missed their appointments (52%) (Table 1). Most failed dental appointments occurred in the age group of 7 to 12 years (53.40%). The second and the third highest occurrences of failed appointments were in the age groups 1 to 6 years and 13 to 18 years respectively (Table 2).

Nearly half of the patients (54.40%) who missed appointments were males, while 45.60% were females (Table 3); 52.0% of the patients’ parents relied on their memory to remember their appointments, 24.8% used their diaries to note down the appointments, 19.7% used their mobile phones as means of reminder, and 3.4% relied on other methods, such as appointment cards and calls from the doctor. Results stated that participants who remembered appointments via memory had the highest no-show rate (p = 0.024) (Table 4). When the reason for missing an appointment was asked, the highest percentage (50%) of the patients missed an appointment due to their exams and parents’ forgetfulness followed by patient being ill on the appointment day (45%) and patient’s symptoms getting better (45%) (Table 5). For canceling an appointment, the highest percentage stated that patient was unwell on the day of the appointment (82%) followed by parent’s commitments toward other work (44%) (Table 6). Approximately two-thirds of the surveyed sample who belonged to the upper middle class showed the maximum no-show rate (58.8%) (Classification of the socioeconomic status was done using Kuppuswamy’s socioeconomic staus scale,1976)^7^ (Table 7); 48.3% of the participants preferred receiving a call from the dentist a day prior to their appointment as a reminder aide, followed by 25.9% preferring the use of appointment cards with date mentioned on it, and 23.8% said that they would want a text message to be sent a day earlier, whereas 2% would want to receive their appointment reminder via e-mails (Table 8).

**Table 1:** **Table 1:** Percentage of patients who have missed appointments and those who have not missed

		*Frequency*		*Valid percent*	
Yes		153		52	
No		141		48	

**Table 2:** **Table 2:** Correlation between the age of no-shows and the missed appointments

*Age range (years)*		*Frequency (no. of children who participated)*		*Frequency (no. of children who missed appointments)*		*Valid percent*		*p-value*	
1-6		95		49		51.60			
7-12		146		78		53.40		0.951	
13-18		52		26		50			

Children in the age group of 7 to 12 years missed the maximum appointments; p-value not significant.

**Table 3:** **Table 3:** Correlation between the gender of the patient and missed appointments

*Gender of child*		*Frequency*		*Valid percent*		*p-value*	
Male		164		54.4		0.089	
Female		130		45.6			

Males missed more appointments compared with females; p-value not significant

**Table 4:** **Table 4:** Percentage of patients remembering appointment date via different methods

		*Frequency*		*Valid percent*		*p-value*	
Memory		153		52.0			
Writing it down in a diary		73		24.8		0.024	
Keeping a reminder in the phone		58		19.7			
Other		10		3.4			

52% of the people who remembered appointment via memory missed the maximum no. of appointments; significant at p < 0.05.

**Table 5:** **Table 5:** Frequency of reasons for failure to keep appointments

*Reason for missing appointment*		*Frequency*		*Valid percent*	
Forgot about the appointment		50		17.0	
Patient too ill to attend		45		15.3	
Patient’s symptoms were better		45		15.3	
Child’s fear of going to the dentist		11		3.7	
Appointment was not with the doctor of choice		11		3.7	
Patient had exams to attend		50		17.0	
Parent’s commitments toward other work		28		9.5	
Other		12		4.1	

Forgetfulness and exams are the major reasons

**Table 6:** **Table 6:** Frequency of reasons for failure to cancel appointments

*Reason for canceling appointment*		*Frequency*		*Valid percent*	
Patient was unwell		82		27.9	
There was long waiting at the clinic		31		10.5	
Child was not in mood for the treatment		27		9.2	
Patient was stuck in traffic		5		1.7	
There was problem with scheduling the appointment		36		12.2	
Financial problem		18		6.1	
Parent’s commitments toward other work		44		15.0	
Other		7		2.4	

Patient unwell is the major reason

**Table 7:** **Table 7:** Correlation between the socioeconomic status of the patients and the missed appointments (according to Kuppuswamy’s classification)

*Socioeconomic status*		*Frequency*		*Valid percent*		*p-value*	
Upper		28		35.7			
Upper middle		216		58.80		0.142	
Upper lower		16		18.80			
Lower middle		34		38.20			

Upper middle-class people have missed more appointments; p-value not significant.

**Table 8:** **Table 8:** Percentage of patients favoring certain appointment confirmation method

		*Frequency*		*Valid percent*	
SMS		70		23.8	
Call from dentist		142		48.3	
Appointment written on card		76		25.9	
E-mail		6		2.0	

Note: Maximum no. of patients preferred a call from the dentist

## DISCUSSION

The goal of this study was to determine the frequency and reasons of missed and canceled pediatric dental appointments and any association with some demographic variables. The sample for this study was drawn from the pediatric dental clinic of a dental college in Navi Mumbai, Maharashtra, India.

In this study, the proportion of missed appointments was found to be 52.0%.

The most common reason for missing an appointment was forgetfulness, which is consistent with the findings of a study by Murdock et al,^8^ Lacy et al,^9^ and AlSadhan.^2^ In our study, forgetfulness was due to the length of the time between scheduling an appointment and the actual appointment date.

The highest percentage of participants still relied on their memory to remember their appointment date, which could be related to high rates of no-shows. It would be reasonable to assume that missed appointment rates could be reduced if patients were advised to use their mobile phones or appointment cards more to record their appointments. The second most common reason for missing an appointment was patient having exams. AlKanderi and AlBader,^10^ in their study said that, “Patients do not keep up their appointments for various personal and logistical reasons, exams being one of them.”

Males were more likely to fail their appointments as compared with their female counterparts which is in accordance with the findings of AlKanderi and AlBader^10^ and AlKanderi and AlBader,^10^ who concluded that male gender is associated with irregular attendance or carelessness toward the treatment and busy work schedule. Contrary to this finding, AlSadhan^2^ stated that females show more failure rates. However, both these studies were conducted in adult patients.

Patient being unwell was the most common reason for canceling an appointment in our study,^11^ which is similar to the findings of Trenouth and Hough, 1991.^12^ Parents commitments toward other work also lead to increase in the rates of canceling and missing an appointment.^11^

Murdock et al^8^ suggested that parent apathy plays a major role in the current burden of missed appointments. Children are dependent on their parents for achieving good oral health care. The concept of “being overloaded in everyday life” explains why parents fail to visit the dental health clinics with their children or to encourage and supervise them, when they can take the responsibility themselves.^11^ As per a study by Lacy et al,^9^ parents themselves lack a tradition of routine dental health care owing to restricted or poor finances or low interest in prioritizing their oral health. Our results suggest that the process of making and keeping clinic appointments is multifactorial rather than the result of a single decision.^9^

Another important finding of why patients miss appointments is patient age. In our study, as the patient age increased, the rate of missed dental appointments decreased significantly. This is in line with the study done by Ismail et al.^6^ Patient age explains that the younger the patient, more the missed appointments due to fear of pain or anxiety, child not in mood for treatment, and child not cooperative. Psychology of the child patient is one of the major issues that must be focused on. Altering the psychology, if deemed necessary, must be done in the first visit itself. The first dental visit is the most important visit not only from a treatment planning point of view but also from a psychological point of view. During the first visit, the dentist should try to understand whether or not the patient has a habit of missing dental appointments or avoiding the dentist. The patient’s logic for doing so must also be clearly understood and psychological alteration should be done according to this.^6^

Education among the parents regarding the importance of oral hygiene and the consequences of missing the appointment on the treatment procedures also play an important role. However, in our study, the effect of education level of parents on children missing their appointments was not found. It must be explained to the parents that dental treatment is not only necessary when pain is present, but is necessary to enhance oral health, which is vital for a better quality of life.

The findings of our study also suggested that patients who missed appointments were of a higher socioeconomic status, which is contrary to the results by Alhamad,^5^ Boyette and Sirois 2011^13^ and Vijayan.^4^ The reasons for high socioeconomic status patients not showing up in our study were:

- No time for parents to accompany their children due to their busy work schedule.
- Difficulties in managing their life situations like job outside home make it difficult to manage extra tasks like dental appointments.
- Lack of parental confidence.

However, other reasons for such people to miss the appointments could be:

- After a hectic day at work, often felt tired and needed a recovery/relaxation.
- Having low priority for dental treatment or regular dental check-ups for their child.
- They would choose to spend money on other things.
- Even if parents wish to take their child, they fail to do so owing to the child’s unwillingness to have dental care.

On asking, most of the people in our study preferred receiving a call from the dentist a day prior to their appointment date as a reminder. According to Vijayan,^4^ verbal human contact with the patients 48 hours before the appointment plays a significant role in the reduction of patient nonattendance. Missing an appointment in the pediatric dental clinic can disrupt the patient’s treatment to a large extent as well as causes a vexing problem for the providers, by limiting the practice efficiency. So, controlling missed and canceled appointments should begin with the patient’s first visit only, where the importance of maintaining the appointment schedule and its effect on treatment outcomes should be clearly communicated to the patient’s parents. Also, the parent should know how and when they should inform the clinic if they were unable to attend.

To overcome these no-show rates, system for scheduling appointments needs to be more flexible, by extending clinic hours, leaving more open slots for walk-ins and incorporating time management in our practice. The use of reminder system, such as telephone reminders (calls or text messages) by staff or by an automated system, can be used to improve the attendance rate, as it has proven to be successful in previous studies by Hashim et al^14^ and Vijayan.^4^ Also, in a study by AlKanderi and AlBader,^10^ the use of SMS text reminders resulted in a statistically significant reduction in the number of failed attendances at appointments. Various other models to reduce the no-show rates are as follows:

- Appointment cards with appointment date mentioned on it
- Appointment date noted on the calendar by the patient
- Preventing booking appointments a long time in advance
- By doing overbooking (Hasvold and Wootton 2011). It is a method of scheduling appointments where clinics book more than the actual number of patients that they can accommodate. It is done with the expectation that some patients might not show up.^15^
- By using automated messaging system (Guy et al),^16^ like Televox solutions, software tools like Lybrate, Practo.
- Sending e-mails a day prior to the appointment
- Providing exit interviews (Guse et al), wherein the patients’ questions are addressed and they are further educated about their future appointments.^17^

Future studies should concentrate on the methods that can reduce or eliminate missed and canceled appointments and encourage patients’ attendance to enhance the treatment outcome in addition to improving the economics and quality of pediatric dental practice.

This study highlights a number of implications for future research. More work needs to be done to engage people who miss appointments into research in a meaningful way.

## CONCLUSION

- Around one half of the patients attending our pediatric dental clinic missed their appointments.
- The highest percentage of the participants relied on their memory to remember their appointments.
- The most frequent reasons for missing an appointment were forgetfulness and exams.
- The most common reasons for canceling an appointment were patient being unwell and parents’ commitments toward other work.
- Maximum number of people said that they would like to receive a call from the dentist a day prior to their appointment date as a reminder aid.
- Patients most likely to fail to keep an appointment is the one who is young, comes from a high socioeconomic status, most of them being males, and have previously broken appointments.

Thus, developing a flexible, easily accessible, interactive appointment with reminder or recall system is highly recommended to overcome the no-shows.

## CLINICAL SIGNIFICANCE

Missed appointments have serious clinical and economic impacts in a pediatric clinic. It deprives another patient from getting an appointment, causing a delay in the treatment of both the patient who got the appointment and the patient who did not get an appointment. This in turn would lead to the development of long waiting lists for dental services as patients who fail to honor appointments will, perhaps, want to be treated later. It disrupts continuity of patient care, delays treatment, and affects doctor-patient relationship, and increases the cost of health care. The nonattendance of clinic appointments impacts negatively on patient care, since patients may miss out on the opportunity to receive treatment for their problems which may later aggravate with time.
